# Ovarian and uterine arteries blood flow velocities waveform, hormones and nitric oxide in relation to ovulation in cows superstimulated with equine chorionic gonadotropin and luteolysis induction 10 and 17 days after ovulation

**DOI:** 10.1186/s12917-023-03692-3

**Published:** 2023-10-13

**Authors:** Elshymaa A. Abdelnaby, Abdulrhman K. Alhaider, Amal M. Abo El-Maaty, Refaat S. A. Ragab, Adel A. Seida, Diya A. El-Badry

**Affiliations:** 1 Department of Clinical Sciences, College of Veterinary Medicine, King Faisal University, P.O. Box 400, 31982 Al-Ahsa, Saudi Arabia; 2 Theriogenology Department, Faculty of Veterinary Medicine, Cairo University, Giza Square, Giza, 12211 Egypt; 3 Animal Reproduction and AI Department, Veterinary Division, National Research Centre, Dokki Giza, Egypt; 4 Artificial Insemination and Embryo Transfer Department, Animal Reproduction Research Institute, Agriculture Research Center, Giza, Egypt

**Keywords:** Cows, Doppler indices, eCG, Ovarian artery, Uterine artery

## Abstract

To investigate the ovarian responses, ovarian and uterine hemodynamics, circulating ovarian hormones, and nitric oxide (NO) with their relations in superstimulated cows. Eight Holstein Friesian dry cows previously synchronized with CIDR underwent rectal Doppler ultrasound scanning and blood sampling after administrating eCG (1500 I.U) on day 10 of the second ovulation (day -5). Cows were treated with 12.5 mg prostaglandin F_2_α (PGF_2_α) on days 10 and 17 after ovulation. Estradiol, progesterone, and NO were measured. Results showed that from ≥ 13 follicles, five follicles ovulated from both ovaries. The ovulated follicles increased antrum colored area and colored area % till day -1. The developed corpora lutea (CLs) attained similar diameter, area, colored area, and colored area % from day 2 till day 15. The peak point of velocity (PSV) of uterine arteries decreased while that of ovarian arteries increased from day -4 to day 0. Both ovarian arteries diameter, resistance index (RI), PSV, end velocity (EDV) and systolic/diastolic ratio (S/D) positively correlated (*P* < 0.0001), but their pulsatility index (PI) negatively correlated (*P* < 0.0001). The uterine arteries PI, RI, PSV, EDV, time average velocity (TAMV) and S/D negatively correlated (*P* < 0.0001) but their diameters positively correlated. Estradiol increased but progesterone decreased from day -5 till day 0. After ovulation, P4 reached maximum values on day 9 and started to decrease till day 19.NO showed one peak on day -3 and another one from day 3 to day 9. Conclusions: Blood flow of ovarian arteries is different from uterine arteries and depended on pre- or post-ovulation.

## Introduction

In cattle, both Follitropin and Pluset were used to induce superovulation with no difference in the proportion of low-responding donors having fewer than five corpora lutea and the recipients did not differ in the non-return rate after transfer of embryos originating from donors protocols [1]. The superovulatory response of cattle to Follitropin was greater when the diameter of the largest follicle exceeded 10mm before superovulation treatment [2]. The responses of cows to equine chorionic gonadotropins (eCG) were also determined [3], and evaluated using Doppler ultrasound [4, 5]. In the bovine estrous cycle, Doppler ultrasonography accessed the preovulatory follicle blood flow [6], and the ovarian and the uterine arteries blood flows [7, 8]. In women, Doppler ultrasound evaluated superovulation protocols for monitoring the uterine artery and the perifollicular blood flows [9]. In mares [10, 11], cows [12],buffalos [13], and small ruminants [14] the uterine and ovarian arteries blood flows were investigated in conjunction with the ovarian response to that directly effects on superovulation.

This study aimed to investigate the ovarian responses, hormonal changes, the dynamics of the uterine and ovarian arterial blood flows and their relation to multiple ovulations in cows stimulated with eCG till after induction of luteolysis on day 10 and 17 to resume their single ovulation.

## Materials and methods

### Animals, treatments and ultrasound scanning

This study was performed in accordance to Institutional Animal Care and Use Committee, Cairo University. The current study used eight (*n*=8) adult, healthy cycling dry Holstein Friesian cows of 3-5 years old, 3.5 BCS, 420 ± 20 kg body weight belonged to the farm of Faculty of Veterinary Medicine, Cairo University. Cows were maintained under the uniform conditions of feeding and management and were kept individually in an indoor paddock with an artificial lightening at night. Cow’s nutritional maintenance requirements composed of commercial concentrated ration and hay with clean water. Ovulation was affirmed by each other day ultrasound examination for two successive ovulations.

This study started from 13^th^ july 2017 by synchronizing estrous cycle using Controlled Internal Drug Releasing device insert for 7 days (CIDR Device, EAZI-BREED™). One day before CIDR removal, 12.5mg of prostaglandin PGF2-analog (LUTALYSE, Upjohn, Egypt) were injected sub-mucosal then the device was removed [15]. After removal of the CIDR devices, cows were examined each other day with Doppler ultrasound to determine the first and second ovulations (day 0). On day 10 after the second spontaneous ovulation (day -5, August 21^st^ 2017) cows received I.M. injections (1500 IU) of equine chorionic gonadotropin (eCG; Pregnecol®, Serum Gonadotropin, 6000 i.u. per vial, Australia), followed by prostaglandin injection (12.5mg, LUTALYSE, Upjohn, Egypt) 48 hours later (day -3). After the multiple ovulations, on day 10 (September 4^th^), and day 17 (September 11^th^), cows were administered 2.5 ml of PGF_2_α-analog (25mg, LUTALYSE, Upjohn, Egypt). Follicles, corpora lutea, ovarian and uterine arteries were examined by Doppler ultrasound from the day of eCG administration (Day -5) till day 19 after multiple ovulations (September 13^th^). Animals were not anaesthetized and/or unconscious during the study.

A trans-rectal pulsed-wave Doppler scanner equipped with 7.5MHz linear-array trans-rectal transducer (EXAGO, made in France) was used by the color and spectral modes. Ovulation was determined by the disappearance of a large dominant preovulatory follicle (>13mm) and corpus luteum development at its location. The last day the dominant follicles were monitored is considered the day of ovulation (day 0) and days after ovulation included days from day 1 to day 19 [16, 17]. The CL diameter was measured throughout the estrous cycle and was divided into growth, static, and regression [18, 19].

The electronic calipers of the ultrasound determined the largest diameter of each follicle or each corpus luteum (CL) per ovary, in addition to ovarian and uterine arteries [17, 20]. The color mode determined the direction of blood flow and the vascularization area, while spectral Doppler measured the peak systolic velocity (PSV), end diastolic velocity (EDV), resistance index (RI) and pulstility index (PI),time average mean velocity (TAMV) and systolic /diastolic ratio (S/D) [21, 22].

### Image analysis

Real-time B mode/color Doppler images were stored in the hard drive using a removable hard disk for blood flow area analyses of follicles and corpora lutea with the ovarian and uterine arterial blood flow spectral Doppler data collection. The follicular and luteal color flows were counted per pixel using Adobe Photoshop CC then each measurement was used to count the selected areas in pixels. Blood flow areas in each follicle wall were measured by outlining a belt circumscribing the anechoic antral cavity of follicle as described using the magnetic lasso tool [23, 24].

### Blood sampling and hormone assaying

Blood samples were collected via jugular vein punctures in plain vacuum tubes of all cows. Serum was harvested and stored at -20°C until hormone assaying. Progesterone (P4) and Estradiol (E2) were analyzed using ELISA kits (competitive type; DRG, Germany). For measuring nitric oxide metabolites (NO), serum samples were mixed with an equal volume of freshly prepared Griess reagent, Nitrite (NO_2_) standards (0–50 mM) were used to determine NOMs concentration as previously measured in mares [25]. The sensitivity of the assay were (0.045 ng/ml, 9.7 pg/ml and 0.225 mmol/L) and the intra- assay and inter-assay coefficients of variation were (5.4 and 9.96, 6.81 and 7.25, 5.3% and 6.9%) for P4, E2 and NOMs, respectively.

### Statistical analysis

Data are presented as mean, standard deviation (SD), and the standard error of the mean (SEM). Repeated measures ANOVA using general linear model was used to study effect of the days from treatment with eCG till ovulation on ovarian follicles and days after ovulation on corpora lutea growth and vascularization, and days before and after ovulation on ovarian and uterine arteries hemodynamics using SPSS software with studying time and treatment interactions [26]. Data are presented in Plots with error bars. Paired sample t-test and pearson correlation coefficients testes were also processed for comparing each pair of follicle/CL area and color areas on both ovaries, ovarian arteries and uterine arteries cross-sectional diameters, RI, PI, PSV, and EDV, TAMV and S/D. Pearson correlation coefficients was also processed to correlate the ovarian hormones and NOMs with every ovarian and uterine arteries cross-sectional (CS) diameters (CS), RI, PI, PSV, EDV, TAMV, S/D and their mean.

## Results

### Follicular responses to treatment with eCG

**Fig. 1:**
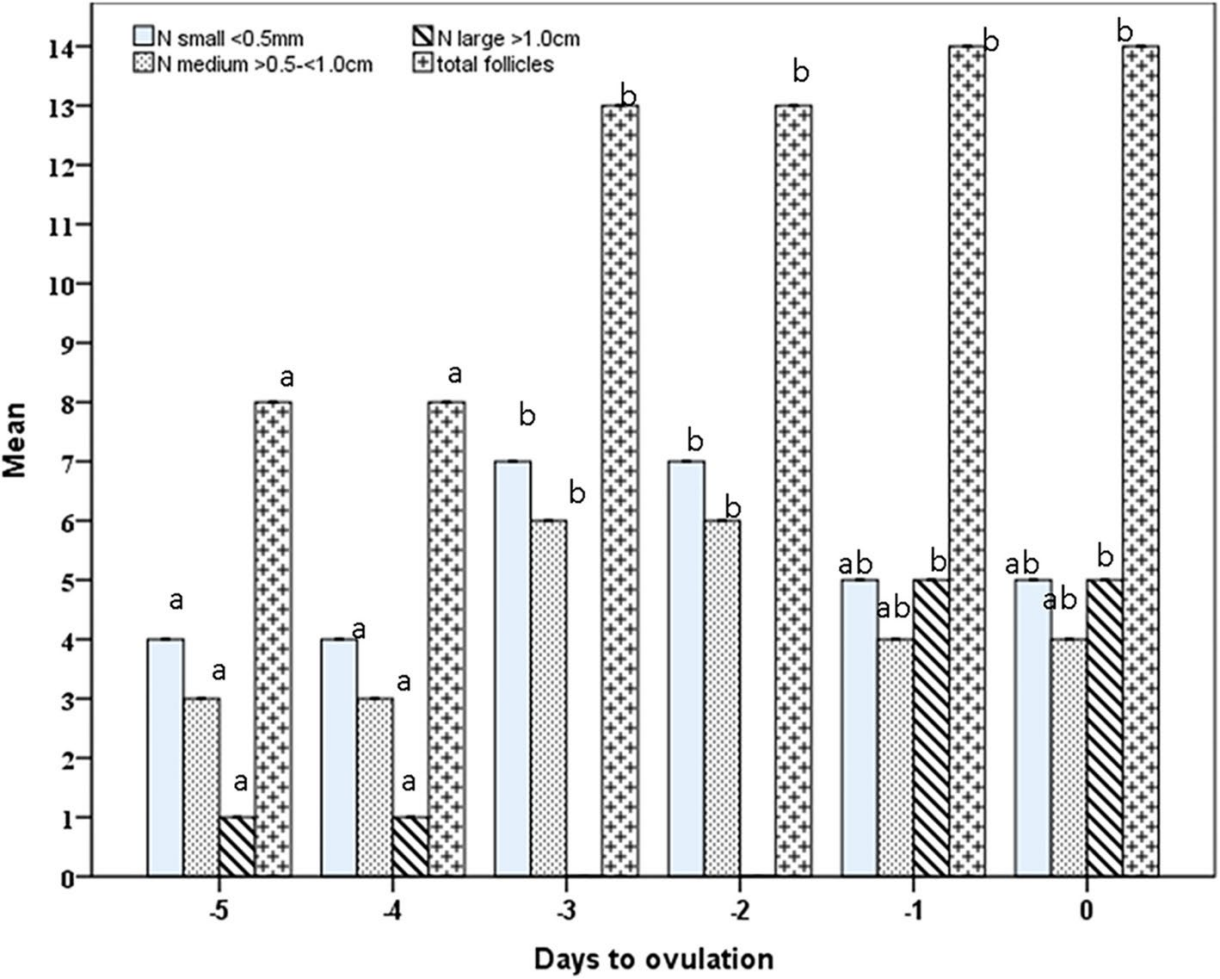
Mean ± SEM number of small, medium, large and total follicles from day of eCG treatment (-5) till ovulation (day 0). Means with different superscripts are significantly different at *P* < 0.05

**Fig. 2:**
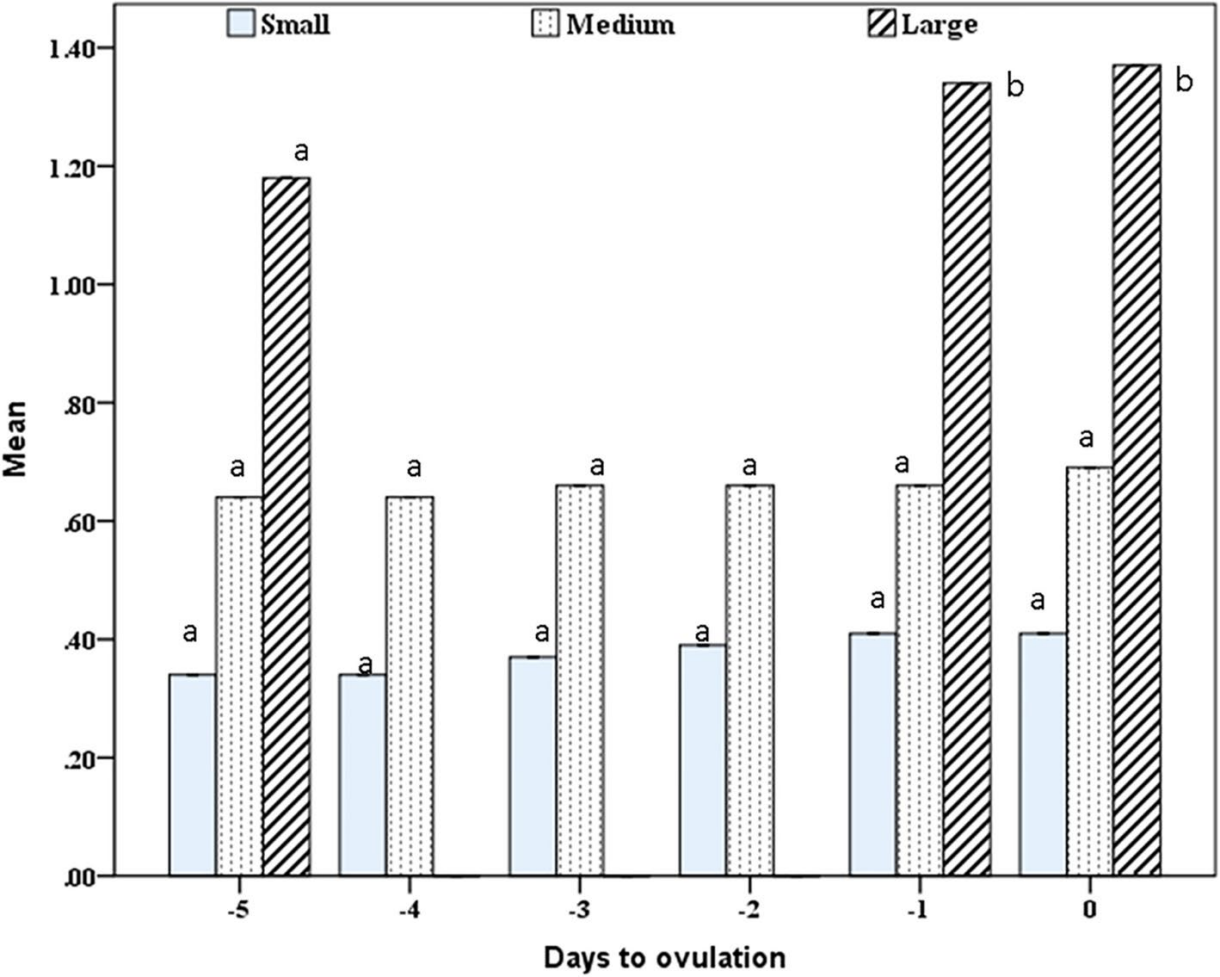
Mean largest diameter of small, medium, and large follicles from day of eCG treatment (-5) till ovulation (day 0). Means with different superscripts are significantly different at *P* < 0.05

**Fig. 3:**
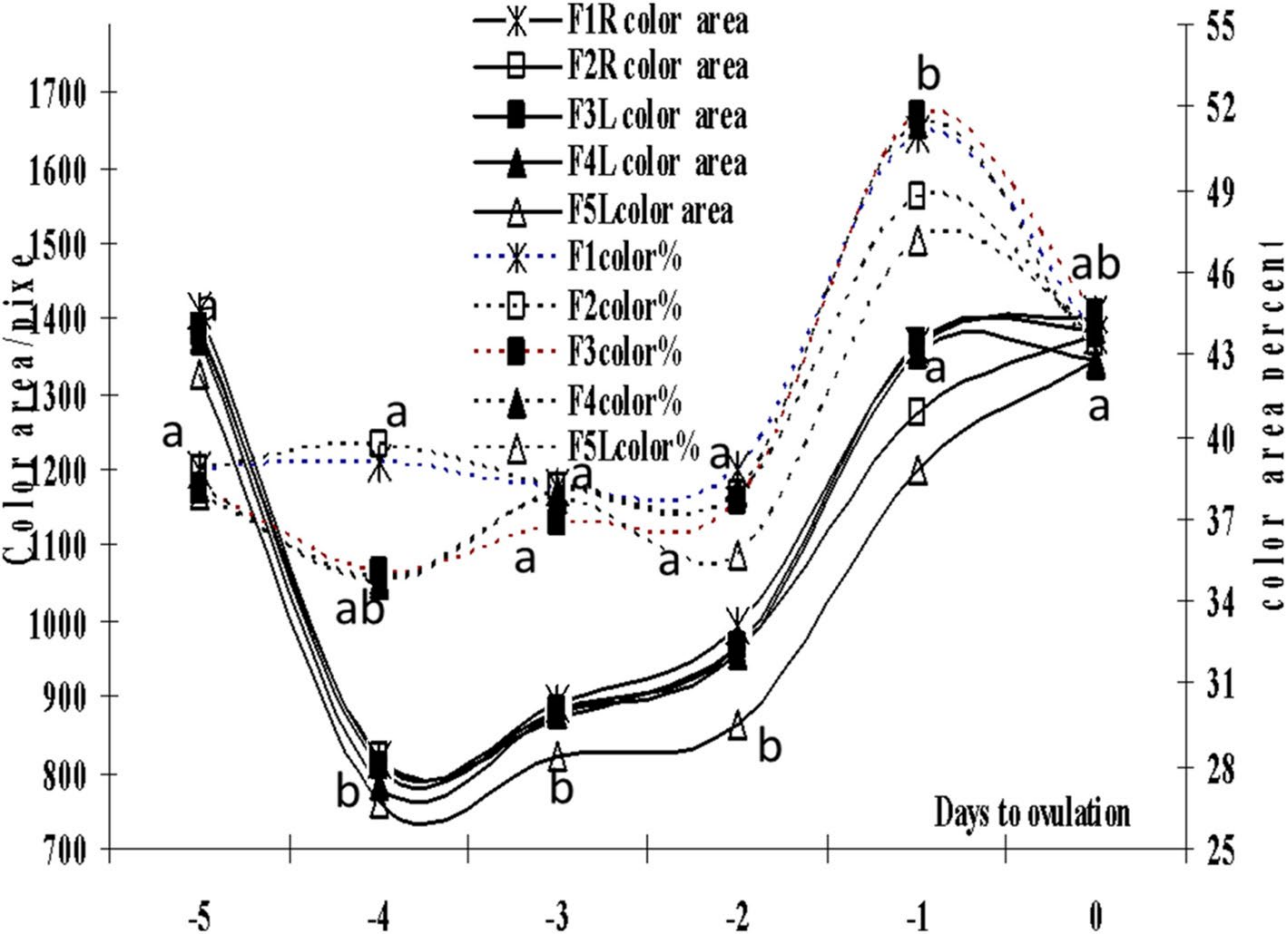
Mean color area/pixel and color area percent of the largest five follicles (F1-5) from day of eCG treatment (-5) till ovulation (day 0). R means on the right ovary, L means on the left ovary. Means with different superscripts are significantly different at *P* < 0.05

The number of small and medium follicles increased (*P*=0.0001) during days -3 and -2 before ovulation (7±0.01, 6 ± 0.01) after being low on day -5 (day of treatment), and day -4 before ovulation (4±0.16, 3±0.10; Fig. 1). The large ovulating follicles increased in number from one follicle on days -5 and -4 to a maximum number (5 ±0.01) on day -1 and day 0 (Day of ovulation). Total follicles increased in number from days -5 and -4 (8±0.01), and stabilized in their number till ovulation (14±0.01; Fig. 1). Small, medium and large follicles diameters/cm increased (*P*=0.0001) linearly to attain their maximum diameters on the day of ovulation (0.41±0.02, 0.69 ±0.01, 1.37±0.02; Fig. 2). The follicle colored area increase (*P*=0.0001) linearly from day -4 to day -1 and slightly declined on day 0 in some of the large follicles (Fig. 3). The colored area % was highest (*P*=0.0001) on the day before ovulation (day -1), and followed the same pattern of the follicle antrum (Fig. 3).

### Corpora lutea (CLs) vascularization

**Fig. 4:**
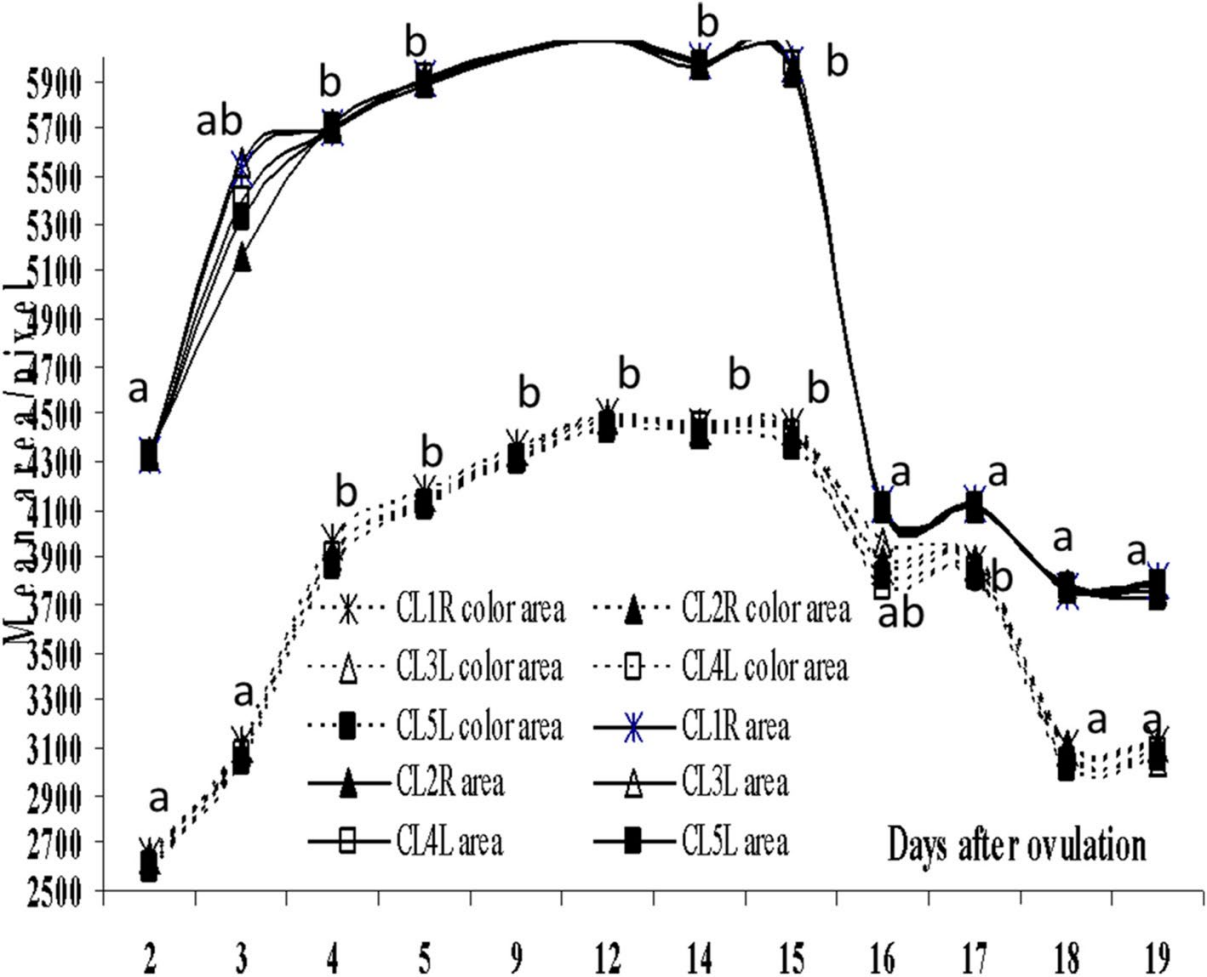
Mean area and color area/pixel of the corpora lutea (CL1-5) from day of ovulation (day 0). R means on the right ovary, L means on the left ovary. Means with different superscripts are significantly different at *P* < 0.05

**Fig. 5:**
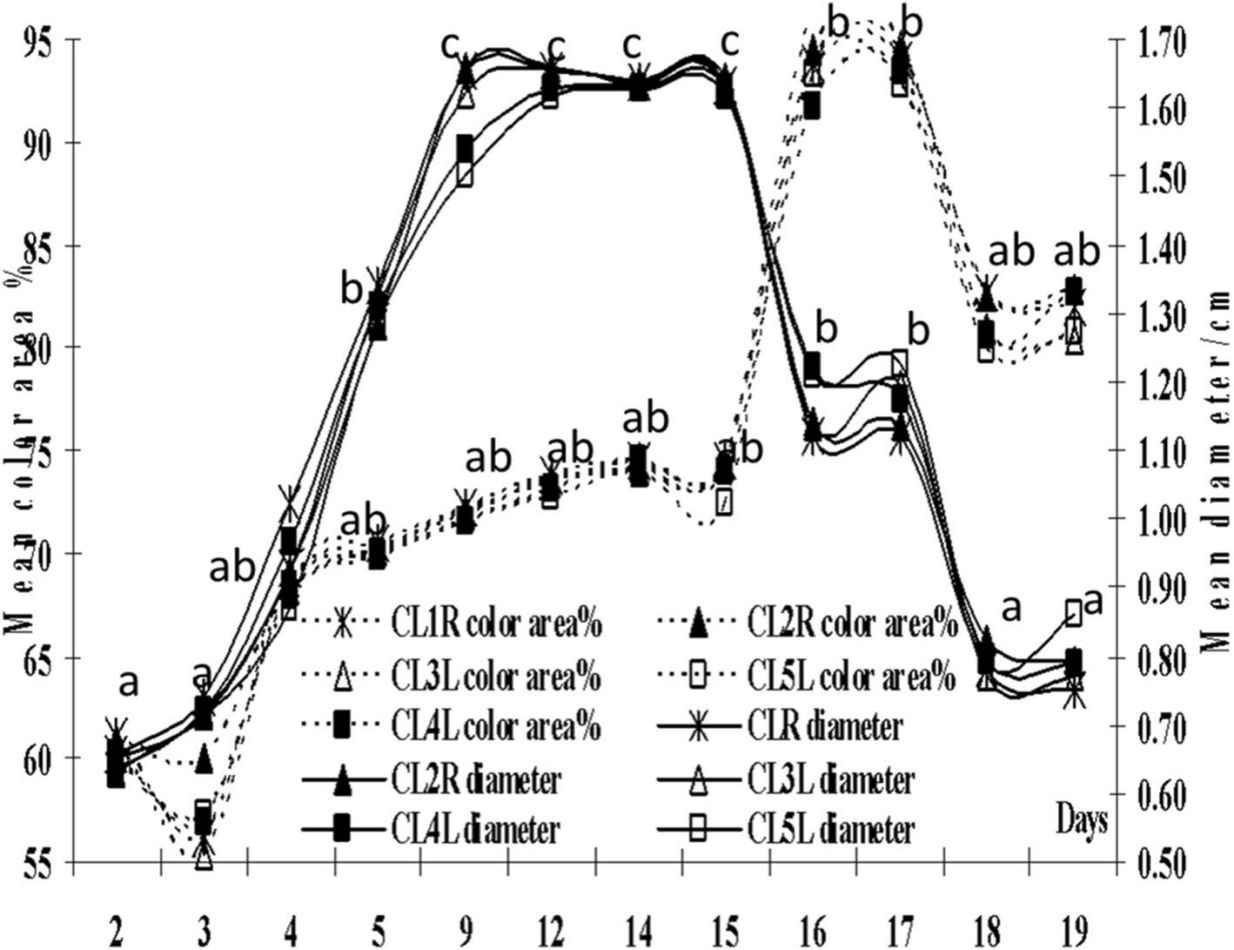
Mean color area% and diameter of the corpora lutea (CL1-5) from day of ovulation (Day 0). R means on the right ovary, L means on the left ovary. Means with different superscripts are significantly different at *P* < 0.05

The growing CLs on both ovaries had the same ascending pattern (*P*=0.0001) of their area and colored area from day 2 till day 15, then both descend till day 19 (Fig. 4). The CLs diameters and colored area % increased (*P*=0.0001) from day 2 till day 15 (Fig. 5). Four days following the prostaglandin F2α administration on day 10, the CLs diameters descended and reached lower values on day 19, whereas, the colored area % increased transiently after on days 16 and 17, and then declined one to two days after the last PGF2 α administration (days 18 and 19). Though the area and the colored area of each pair of Follicle/CL varied (*P*=0.0001), but their areas only correlated (*P*< 0.05, Table 1).

**Table 1:** The comparison between each follicle and its corresponding CL mean area and color area, their ratio and their correlations on the right and left ovaries

Follicles/CLs	Mean	Standard Deviation	Standard Error	Sig	F/CL ratio	r	*P*-value
F1R area	1884.68	637.038	65.017	0.0001	2.78	0.238	0.020
CL1R area	5235.86	863.323	88.113				
F1R color area	1009.69	164.674	16.807	0.0001	3.76	0.010	0.920
CL1R color area	3762.58	607.746	62.028				
F2R area	1827.50	636.215	64.933	0.0001	2.85	0.247	0.015
CL2R area	5208.97	860.704	87.845				
F2R color area	976.07	167.153	17.060	0.0001	3.83	-0.041	0.694
CL 2 R color area	3745.83	602.722	61.515				
CL3 L area	1901.72	682.217	69.629	0.0001	2.75	0.200	0.050
F3L area	5233.63	861.717	87.949				
F3L color area	991.44	163.913	16.729	0.0001	3.78	0.003	0.974
CL3 L color area	3742.06	612.254	62.488				
F4L area	1875.06	661.209	67.484	0.0001	2.78	0.234	0.022
CL4 L area	5216.31	866.495	88.436				
F4L color area	957.73	196.492	20.054	0.0001	3.89	0.086	0.407
CL4 L color area	3719.96	609.593	62.216				
F5 area	2036.88	523.196	53.398	0.0001	2.55	0.230	0.024
CL5 L area	5205.49	863.752	88.156				
F5L color area	910.05	179.848	18.356	0.0001	4.07	-0.031	0.762
CL5 L color area	3700.06	607.768	62.030				

### Ovarian arteries blood flow

**Fig. 6:**
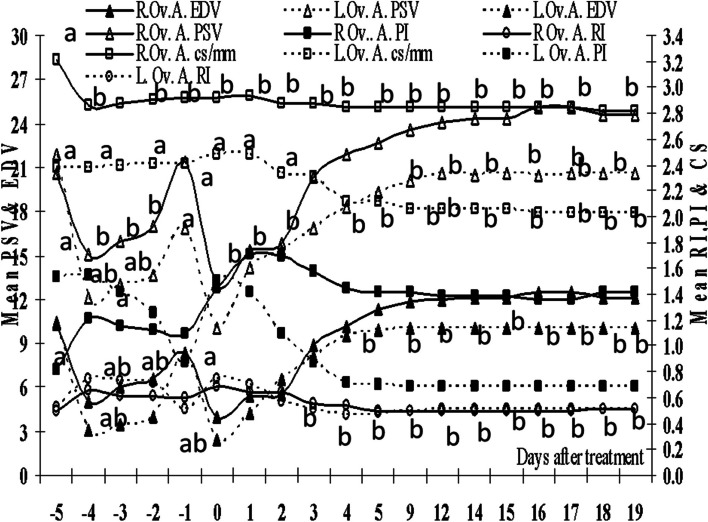
Mean right ovarian (R.Ov.) and left ovarian (L.Ov.) arteries cross section diameter (CS/mm), RI (Resistance index), PI (Pulsatility index), PSV (Peak systolic velocity) and EDV (End diastolic velocity). Means with different superscripts are significantly different at *P* < 0.05

In contrast to the lower diameter of the left ovarian artery but their diameters correlated significantly (*P*=0.0001, Table 2). The right ovary (previously ipsilateral to the single ovulation) produced two ovulating follicles and developed two corpora lutea, and the left one produced three ovulating follicles and developed three corpora lutea. Though both ovaries responded (*P*=0.0001) to superovulation with eCG, produced ovulating follicles, and developed corpora lutea but their diameters and blood flow varied during the treatment (Fig. 6),but the right ovarian artery had a higher blood flow as expressed by PSV and EDV.

**Table 2:** The comparison between the mean, the standard deviation (SD) and the standard error (SE) of the right (R) and left (L) ovarian artery (Ov. A.) diameter (CS), blood flow RI, PI, PSV, EDV, AMV, and S/D, the correlations (r) between the right and left ovarian arteries CS, RI, PI, PSV, EDV, TAMV and S/D, and the correlation of the mean ovarian arteries CS, RI, PI, PSV, EDV, TAMV, and S/D with estradiol (E2), progesterone (P4), and nitric oxide metabolites (NOMs)

Ovarian artery	Mean	SD	SE	Sig	r	*P*-value	E2	P4	NOMs
R.Ov. A. CS	2.89	0.090	0.008	0.0001	0.531	0.000	0.64^a^	-0.42^a^	-0.02
L.Ov. A. CS	2.25	0.170	0.015						
R Ov.A. PI	1.35	0.225	0.019	0.0001	-0.304	0.000	0.45^a^	-0.38^a^	-0.36^a^
L. Ovv. A. PI	1.02	0.351	0.031						
R Ov. A. RI	0.57	0.069	0.006	0.015	0.835	0.000	0.72^a^	-0.65^a^	-0.23^a^
L. Ov. A. RI	0.58	0.104	0.009						
R.Ov. A. PSV	20.04	30.942	0.348	0.0001	0.923	0.000	-0.75^a^	0.62^a^	0.13
L.Ov. A. PSV	17.10	30.569	0.3157						
R.Ov.A. EDV	8.90	20.946	0.260	0.0001	0.946	0.000	-0.76^a^	0.65^a^	0.16
L.Ov.A. EDV	7.51	20.939	0.259						
R.Ov.A.TAMV	8.50	10.764	0.156	0.0001	0.178	0.044	-0.63^a^	0.48^a^	0.16
L.Ov.A. TAMV	10.62	30.769	0.333						
R.Ov.A. S/D	2.37	0.399	0.035	0.0001	0.846	0.000	0.70^a^	-0.61^a^	-0.21^b^
L.Ov.A. S/D	2.57	0.794	0.070						

^a^Correlation is significant at the 0.01 level (2-tailed)

^b^Correlation is significant at the 0.05 level (2-tailed)

### Uterine arteries blood flow

**Fig. 7:**
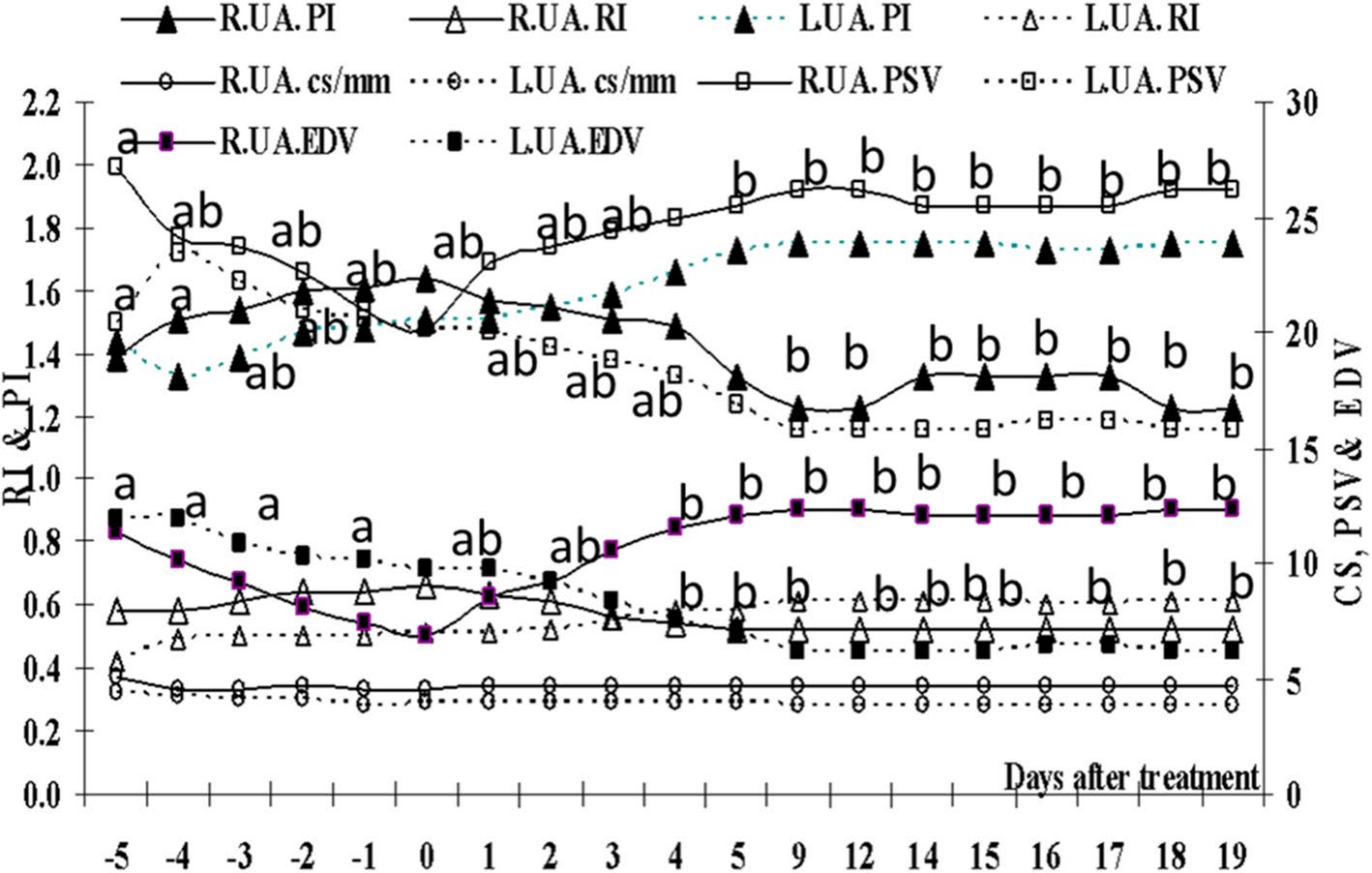
Mean right (R.U.A.) and left (L.U. A.) uterine arteries cross section diameter (cs/mm), RI, PI, PSV cm/sec and EDV cm/sec. Means with different superscripts are significantly different at *P* < 0.05

The diameters and blood flow measures of both uterine arteries are affected (*P*=0.0001) by days after treatment (Fig. 7). The right uterine artery had a higher cross section diameter and PSV along the study, with higher RI, PI and lower EDV only from the start of treatment till day 2. Following treatment with eCG, a marked decrease in the right uterine artery (R.U.A.) diameter was noticed on day -4 then peaked on day -2, declined on day -1 then ascended till day 12, then maintained a slight decrease till day 19. The left uterine artery (L.U.A.) diameter descended directly after treatment till day -1 then started increasing till day 5, after that it declined and maintained nearly the same value till the end of the study. The PSV of the R.U.A. declined after treatment then ascended reaching high values on days 9 and 12 and re-ascended again reaching their previous maximum value. The PSV of the L.U.A. increased sharply for one day then gradually decreased till day 9 and maintained these minimum values till day 19 except for days 16 and 17. The EDV of the left one started decreasing from day -4 reaching minimum values on day 9 and maintained the same low values till day 19 except for days 16 and 17. After treatment, the RI of the right uterine artery increased from day -4 till reaching a peak value on Day 0 then declined reached lowest value on day 5 and kept nearly this value till day 19. The L.U.A. RI increased directly after treatment till day 5 then stabilized for one day and maintained nearly the highest values from day 12 till day 19. The R.U.A. PI increased after treatment till Day 0 then descended till reached a minimum value on days 9 and 12,. In contrast, the L.U.A. started increasing from day -4, reaching high value on day 9 then maintained nearly the same value till day 19.

The diameters of the right uterine artery was higher (*P*=0.0001) than the left one with a marked relation (Table 3). Though the L.U.A. had significantly (*P*=0.0001) higher RI, PSV, EDV, TAMV and S/D (Table 3), but their PI was low and all of them were correlated negatively (*P*=0.0001).

**Table 3:** The comparison between the right (R) and left (L) uterine arteries (U.A.) mean diameter (CS), blood flow RI, PI, PSV, EDV, TAMV and S/D, the correlations (r) between the right and left uterine arteries CS, RI, PI, PSV,EDV, TAMV and S/D, and the correlation of the mean uterine arteries CS, RI, PI, PSV,EDV, TAMV, and S/D with peripheral estradiol (E2), progesterone (P4), and nitric oxide metabolites (NOMs)

Uterine artery	Mean	SD	SE	Sig	r	*P*-value	E2 pg/ml	P4ng/ml	NOMs μmol/L
R.U A. CS	4.65	0.126	0.011	0.0001	.51	0.000	-.29**	.45**	.07
L.U A. CS	3.99	0.147	0.013						
R.U A. PI	1.44	0.141	0.012	0.0001	-.77	0.000	.32**	-.44**	.097
L.U A. PI	1.59	0.141	0.0123						
R.U A. RI	.57	0.050	0.004	0.001	-.77	0.000	.41**	-.56**	-.05
L.U A. RI	.54	0.056	0.005						
R.U A. PSV	24.36	1.879	0.166	0.0001	-.57	0.000	-.09	.24**	.01
L.U A. PSV	18.74	2.441	0.216						
R.U A.EDV	10.34	1.863	0.165	0.0001	-.67	0.000	-.35**	.49**	.01
L.U A.EDV	8.59	2.067	0.183						
R.U A.TAMV	9.80	1.014	0.089	0.0001	-.63	0.000	-.24**	.34**	-.09
L.U A.TAMV	6.48	0.966	0.085						
R.U A.S/D	2.41	0.291	0.026	0.001	-.74	0.000	.58**	-.68**	-.03
L.U A.S/D	2.24	0.264	0.023						

Standard error of the mean (SE), Standard deviation (SD)

^**^Correlation is significant at the 0.01 level (2-tailed)

^*^Correlation is significant at the 0.05 level (2-tailed)

### Hormonal and nitric oxide changes

**Fig. 8:**
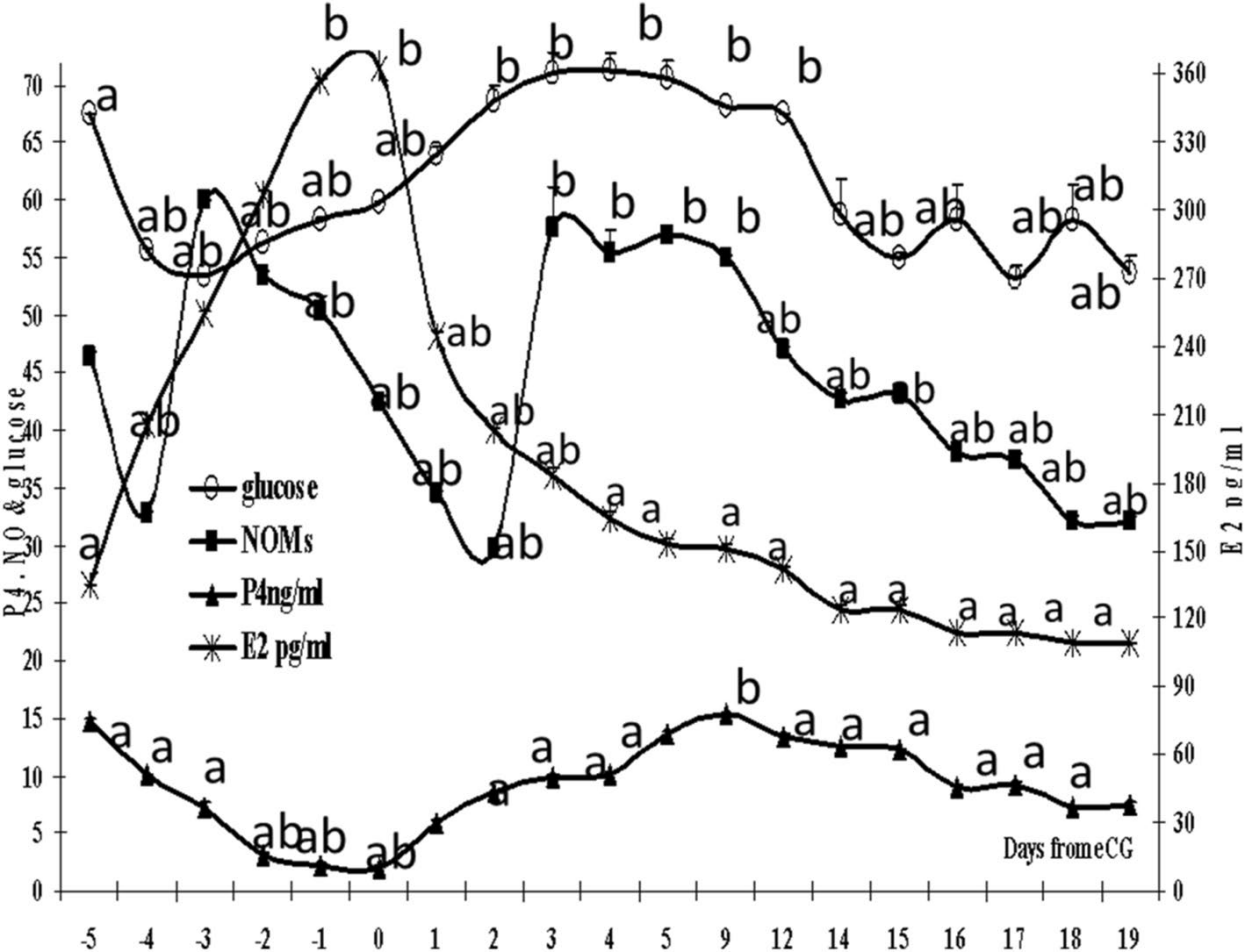
Mean estradiol level(E2, pg/ml), progesterone level (P4, ng/ml), nitric oxide metabolites level (NOMs, μmol/L), and glucose level mg/dL with error bars. Means with different superscripts are significantly different at *P* < 0.05

Once eCG administered, E2 levels ascended reaching their peak on day 0 (*P*=0.0001) then declined in a linear pattern reaching low concentrations from day 14 to day 19. The NOMs (*P*=0.0001) peaked for one day before ovulation (day -3) and form Day 3 to Day 9 after ovulation. Then the progesterone increased reaching their peak values on day 9 (Fig. 8).

Estradiol (E2) had significant (*P*=0.0001) positive correlations with the R. Ov. A. RI and S/D, the L. Ov. A. RI, PI and S/D, the R.U.A. PI, RI, and S/D and the L.U.A. PI, RI, and S/D, but had negative significant (*P*=0.0001) correlations with the R. and L. Ov. A. PSV and EDV (Table 2) and R. and L. U. A. EDV and cross section diameter (Table 3).

While P4 showed positive significant (*P*=0.0001) correlations with R. Ov. A. PSV and EDV but had negative correlations with RI and S/D. in addition P4 had negative correlations with the L.Ov.A. CS, RI, PI, and S/D but had positive correlations with PSV, EDV and TAMV. P4 attained positive correlations with R.U.A. CS, PSV, EDV and TAMV. P4 correlated negatively with the L.U.A. PSV, EDV, and TAMV but positively with CS, RI, PI, and S/D.

NOMs had significant (*P*=0.0001) negative correlations with the R. Ov. A. PI, RI and S/D, positive ones with non-significant with TAMV and EDV but tended to correlate with PSV (*P*>0.05).

## Discussion

This current study found a significant effect of the days after the induction of superovulation using eCG on the number and diameter of ovarian follicles of cows. In comparison to our previous study on the same cows during their single ovulation, only the dominant follicles started growth from 3 mm and reached a diameter of 1.37 cm [8]. In agreement with our study, on the day of superstimulation with eCG, all cows had either one large dominant follicle or a corpus luteum, but 3 and 12 days from the ovarian stimulation, cows had more than 17 follicles ≥0.5cm and mature corpora lutea [5]. In comparison to our results, the higher ovarian response after eCG means the higher dose of eCG [5]. Two days after eCG, more small and medium follicles recruited and stand still for one more day (day -2) till the deviation of the largest ones. Similarly, ovulation in the same cows, the diameter and the vascularization color area of ovulatory follicle increased one day earlier (day -4) then reached highest on day 0 [8].

After ovulation, all the developed corpora lutea (*N*=5) kept the same color area till day 19. The CLs irresponsive to PGF_2_α 10 days post ovulation in the current study may also refer to the presence of more dilated capillaries in the bovine CLs in response to eCG treatment on day 6 six after ovulation [27]. Contrary to beef cows' synchronised ovulation, the presently stimulated cows displayed weak but substantial correlations between the area of each pair of follicles and corpus luteum on either ovary, showing that the follicular vascularity increased CL blood flow and progesterone production [28]. Similar to the single ovulation where the ipsilateral (Right) ovarian artery had larger diameter along the estrous cycle [8], the same super-stimulated cows ovarian artery ipsilateral to the fewer follicles (previously ipsilateral) had larger diameter compared to the other one ipsilateral to the more follicles. Though the ipsilateral ovarian artery RI during single ovulation was lower than the contralateral one till day of mono-ovulation [8], but when multiple ovulations was induced, the RI of the ipsilateral to the fewer follicles were higher than that of more follicles till day 2 after ovulation. In contrast to the single ovulation [8], where the ipsilateral ovarian artery obtained lower PI along the estrous cycle indicating higher blood flow, the PI of the ovarian artery ipsilateral to the ovary with two ovulating follicles was higher than those of the ovary with three ovulating follicles from the day of treatment with eCG till day 1, the mean PI decreased and did not change until day 12 and the absolute increase in blood flow was similar for both arteries and no correlations were observed between the blood flow volume, either the number of follicles >5mm or the developed corpora lutea on the same side [3, 5]. Contrary to our findings, super-stimulated mares [10] and women [29] did not show a difference in the blood flow volume and PI of the two ovarian arteries, but the rise in ovarian blood flow volume with the increase in ovarian follicle number is consistent with our findings. Despite the fact that in super-stimulated mares, the volume of ovarian blood flow did not correspond with estradiol [10]. Just before the ovarian stimulation, the ovary with fewer ovulated follicles (previous ipsilateral) had higher blood flow volume and lower ovarian artery PI only during the follicle growth and decreasing PI till day after ovulation [3], and this disagreement between the current study and some studies may refer to fewer evaluation days [3, 5].

Endometrial perfusion was higher ipsilateral to the mature CL during the normal estrous cycle of Angus cows compared to the contralateral on day 12 [30], but the increase in the previously ipsilateral uterine artery blood flow of our study having fewer CLs blood flow compared to that having more CLs is indicating that the blood flow depends on the previously ovulating ovary and not on the CLs number. In this study, both ovaries produced large ovulating follicles and all the five large follicles ovulated but the right ovary (previously ovulating) had fewer ovulating follicles than the left one indicating that ovarian arteries blood flow is not dependable on the number of ovulating follicles. Different from the PSV, EDV, S/D and diameters, the mean RI of the right uterine artery ipsilateral to the fewer follicles was higher than the left one ipsilateral to the large follicles and both increased from the time of eCG administration till day 0 (ovulation), then from day 4 after ovulation, the RI of the left uterine artery was higher than that of the right one. The same cows during their non induced ovulation had also maximum uterine artery RI on the day of ovulation but the ipsilateral uterine artery kept a lower RI during the preovulatory period [8]. Regardless the day from eCG treatment in our study, the previous ipsilateral uterine artery attained higher blood flow expressed by a lower PI, higher diameter, PSV and EDV, another reports found no differences between the two uterine arterial blood flows and pooled their blood flow volume during analysis [5, 10]. Contrary to the normal estrous cycle where uterine artery PI of Sahiwal cows was higher on day 10 than on day -1 and day 0 [7], and that of cyclic Friesian cows increased from day -2 till day 0 [8], the right uterine artery PI of the currently stimulated cows with eCG increased till day of ovulation and decreased thereafter and that of left uterine artery increased from day -4 and kept increasing till the end of the study. The RI of uterine arteries was considerably lower, while the PI was substantially elevated during diestrus compared to estrus and ovulation in Sahiwal cows [7]. The right uterine artery blood flow was shown to rise after ovulation in this study, and both ovarian arteries were found to increase before and after ovulation as well, with the exception of days 0 to 2 when cows' uterine diameter, PSV, and EDV increased while their PI and RI decreased [4]. Moreover, the association of uterine blood flow volume with estradiol in superstimulated mares [10] expressed by negative RI and PI. The association of increased ovarian blood flow with the increased estradiol observed in cows [5] related to vasodilatory effects of estradiol that are mediated by NO [31]. Estrogen may stimulate release of NO from vascular cells by mechanisms that dependent on gene [32, 33].

Similarly, the peak of increased nitric oxide during multiple follicle growth development associated with increased estradiol and ovarian blood flow while another peak of NO associated with the increased uterine blood flow were reported in mares [25], and jennies [26, 34].

## Conclusions

The synchronization method and the dose of eCG determine the ovarian response. The ovarian blood flow is higher during multiple follicle development but the uterine blood flow is higher during corpora lutea development. The vasodilatation mechanisms of Nitric oxide are mediated by increased estrogen. It was concluded that corpora lutea developed after ovarian stimulation required more than one dose of the normal luteolytic dose for the induction of luteolysis. In addition to it was found that the ovarian arteries blood flow is higher during follicular phase and is not dependable on the number of ovulating follicles.

## Data Availability

All datasets generated and/or analysed during the current study are included in this published article.

## Abbreviations

CIDR: Controlled internal drug release device
CL: Corpus luteum
CLs: Corpora lutea
E2: Estradiol
eCG: Equine chorionic gonadotropin
EDV: End diastolic velocity
NO: Nitric oxide
NO_2_: Nitrite
NOMs: Nitric oxide metabolites
P4: Progesterone
PI: Pulstility index
PSV: Peak systolic velocity
RI: Resistance index
S/D: Systolic /diastolic
SD: Standard deviation
SEM: Standard error of the mean
TAMV: Time average maximum velocity
